# Suicidal ideation, depressive experiences, and resilience among adolescents living with HIV in rural Ghana: a qualitative exploration of psychological experiences and coping

**DOI:** 10.3389/fpubh.2026.1972968

**Published:** 2026-10-30

**Authors:** Dorothy Serwaa Boakye, Samuel Adjorlolo

**Affiliations:** 1 Department of Health Administration and Education, University of Education, Winneba, Ghana; 2 Department of Mental Health Nursing, School of Nursing and Midwifery, University of Ghana, Accra, Ghana

**Keywords:** adolescents, coping strategies, HIV/AIDS, mental health, psychological experiences, stigma

## Abstract

**Background:**

Adolescents living with HIV/AIDS (ALHIV) in sub-Saharan Africa face significant mental and emotional burdens compromising treatment adherence and quality of life. Despite growing evidence of psychological morbidity, the subjective experiences, stigma mechanisms, and coping processes of ALHIV in rural Ghanaian contexts remain poorly understood. This study addressed these gaps guided by Lazarus and Folkman's (1984) Stress and Coping Theory and Link and Phelan's (2001) stigma framework, enabling an integrated analysis of how HIV-related stressors are appraised, how stigma depletes coping resources, and why specific psychological outcomes arise.

**Methods:**

A qualitative descriptive design was employed. Data were collected from 12 adolescents living with HIV through two focus group discussions and three in-depth interviews at Atua Government Hospital, Lower Manya Krobo District, Ghana, via purposive sampling and analysed using Braun and Clarke’s (2017) six-step reflexive thematic analysis.

**Findings:**

Five themes emerged. Emotional and mental distress, encompassing suicidal ideation, fear of death, and depressive tendencies, reflected catastrophic primary appraisal of HIV as an irreversible existential threat. Multi-dimensional stigma, encompassing family-based, religious, and community/peer forms, depleted coping resources at every relational level; religious stigma was identified as a distinct form operating through perceived divine authority, a culturally specific pathway through which Link and Phelan’s existing stigma mechanisms (labelling, stereotyping, and status loss) operate, rather than an extension of the framework itself. Social isolation represented both a consequence of depleted secondary appraisal resources and a self-perpetuating cycle further limiting them. For some participants, fatalistic appraisal of treatment futility appeared to inform reduced motivation to adhere to antiretroviral therapy, one plausible coping-related interpretation among several, alongside structural and relational barriers also documented in the data. Coping mechanisms including religious practices, family connection, and personal resilience mapped onto emotion-focused, meaning-focused, and problem-focused coping respectively; religious contexts functioned paradoxically as both the most potent stigma source and the most widely accessed coping resource.

**Conclusion:**

Findings suggest that ALHIV in this setting encounter HIV through catastrophic appraisal, multi-level stigma, and severely depleted coping resources. These findings suggest that effective interventions should address cognitive appraisal, family systems, and religious contexts simultaneously, rather than targeting symptoms or adherence behaviours in isolation.

## Graphical abstract

**Figure:**
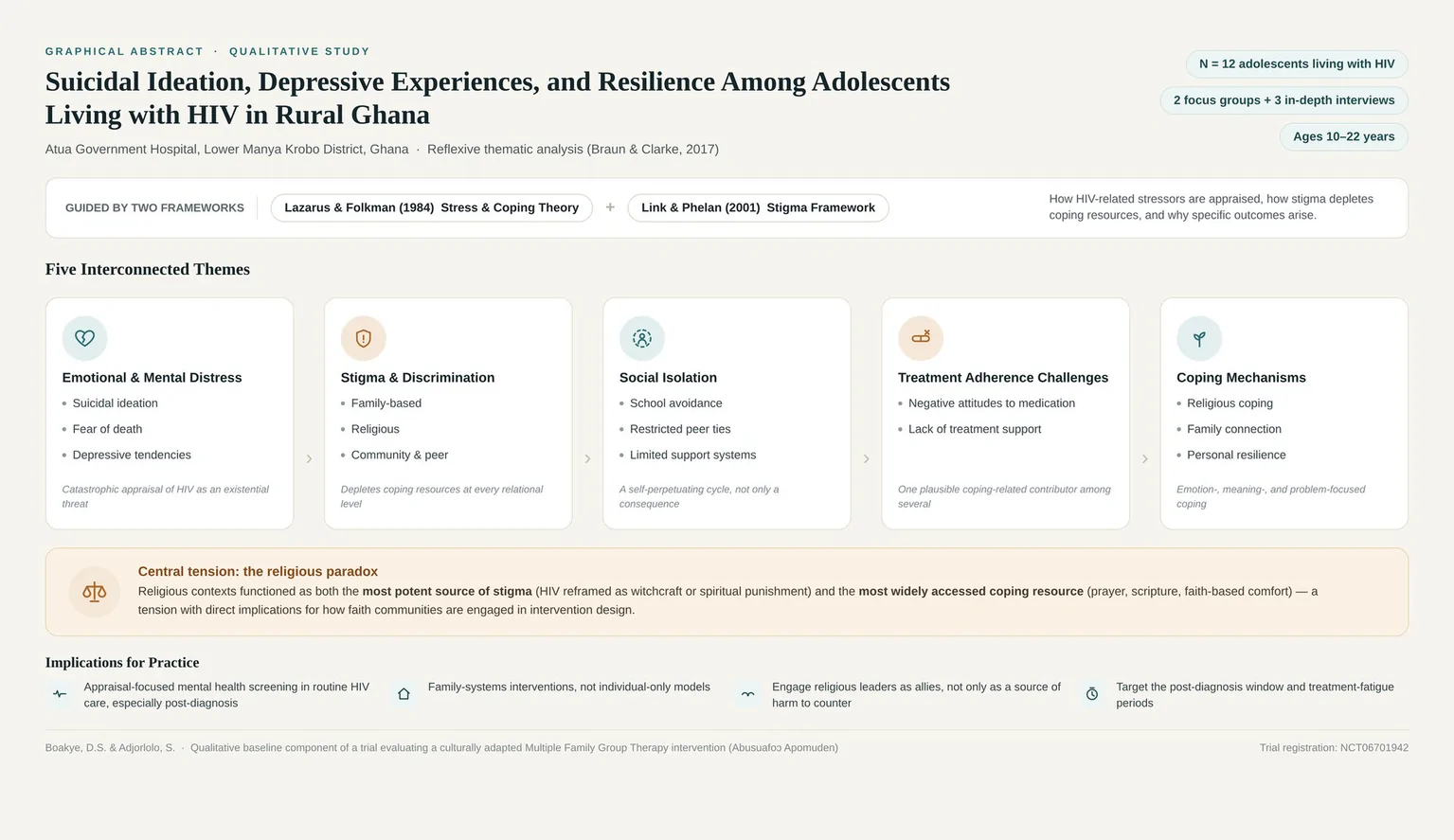

## Introduction

Adolescence represents a critical developmental period characterised by significant physical, cognitive, and psychosocial changes (1). For adolescents living with HIV/AIDS (ALHIV), this already demanding life stage is further complicated by the burden of managing a chronic, stigmatised health condition, creating a distinctive set of mental and emotional hurdles that significantly impact quality of life, treatment adherence, and long-term health outcomes (2).

The burden of adolescent HIV is disproportionately concentrated in sub-Saharan Africa, accounting for approximately 85% of the global adolescent HIV burden, with an estimated 1.55 million adolescents aged 10 to 19 years living with the condition (3, 4). In Ghana, approximately 18,000 children aged 0 to 14 years were living with HIV as of 2023 (5). ALHIV in sub-Saharan Africa experience depression rates two to three times higher than their HIV-negative peers, with prevalence estimates ranging from 14 to 69% (6–8), and 15 to 17% report suicidal ideation (9, 10). These challenges significantly compromise HIV management, as ALHIV with depression are more likely to demonstrate poor treatment adherence (11), a relationship particularly pressing in Ghana, where adolescents achieve markedly lower rates of viral suppression than adults (12). Family-based interventions such as Multiple Family Group Therapy (MFGT) have demonstrated promise in addressing these dimensions (13); however, their cultural adaptation for Ghanaian adolescents requires contextually situated understanding of the specific psychological challenges, stigma mechanisms, and coping processes characterising this population’s lived experience.

Despite this evidence, four critical gaps persist. First, few studies have employed qualitative methods to capture the subjective experiences of ALHIV (2), and the existing literature is weighted heavily towards psychopathology and risk, with comparatively little attention to protective factors, family resilience, and strengths-based adaptation (14). Whilst quantitative studies document prevalence rates (15–17), they do not illuminate how adolescents make meaning of their condition, what emotional challenges they face, what coping strategies and sources of resilience they draw upon, or what support they find beneficial, evidence indispensable for culturally adapting an MFGT programme. Second, existing research has been geographically narrow, concentrated in Western or urban African settings, creating a knowledge gap regarding adolescents in rural or semi-urban areas such as the Lower Manya Krobo District, where stigma manifestations, cultural beliefs, and resources differ substantially from well-studied contexts (18, 19). Third, specific stigma mechanisms, particularly religious stigma, remain inadequately investigated, despite evidence that ALHIV in sub-Saharan Africa frequently report stigma related to spiritual beliefs (20) and that religious institutions shape moral frameworks in Ghana in ways directly relevant to HIV-related psychological distress (21). Fourth, whilst educational disruption is established, with ALHIV in sub-Saharan Africa 60% more likely to experience school non-attendance than uninfected peers (22) and Ghanaian adolescents missing school at least once monthly due to HIV-related issues (23), the psychological mechanisms driving disengagement remain underexplored.

This qualitative study addresses these gaps by exploring the lived experiences of ALHIV in the Lower Manya Krobo District, centring their voices to provide a contextualised understanding of their psychological journeys. Conducted as a baseline component of a randomised controlled trial (NCT06701942) evaluating a culturally adapted MFGT intervention for improving mental health and ART adherence among ALHIV and their caregivers, this study provides a descriptive account of adolescents’ pre-intervention psychological experiences, stigma, and coping strategies, forming the qualitative baseline component of the trial.

### Theoretical frameworks

The primary analytical lens is Lazarus and Folkman's (24) Stress and Coping Theory, which explains how psychological outcomes are shaped not by the objective severity of a stressor but by cognitive appraisal: whether it is perceived as a threat, harm, or challenge (primary appraisal), and what coping resources are judged as available (secondary appraisal). Coping responses are conceptualised as problem-focused, directed at modifying the source of stress, or emotion-focused, directed at regulating the resulting distress. This framework suits the study of ALHIV because it accounts for variation in psychological outcomes among adolescents facing the same stressor, accommodates both adaptive and maladaptive responses, and as a transactional model remains sensitive to the contextual factors central to this study: family support, community stigma, and religious beliefs.

Complementing this, the study draws on Link and Phelan's (25) modified stigma framework, grounded in Goffman's (26) concept of spoiled identity. Link and Phelan conceptualise stigma as a convergence of five interrelated components: labelling (identifying a human difference as salient), stereotyping (associating that label with undesirable characteristics), cognitive separation (constructing an us-versus-them distinction), status loss (devaluing the labelled person’s social standing), and discrimination (enacting that devaluation through exclusionary behaviour). These processes operate at individual, relational, and structural levels, and are particularly relevant to Ghana, where stigma is enacted through family systems, religious institutions, and community structures through culturally specific mechanisms.

Together, the frameworks provide a coherent analytical architecture. Lazarus and Folkman's (24) theory illuminates the psychological mechanisms through which HIV-related stressors produce emotional distress, treatment non-adherence, and variable coping responses, whilst Link and Phelan's (25) framework explains how multi-level stigma processes deplete the coping resources that secondary appraisal depends upon. In combination, they move the analysis beyond documenting what adolescents experience to explaining why specific outcomes arise and through what mechanisms, generating findings that are theoretically grounded and actionable for intervention design.

## Methods

### Study setting

This study was conducted at Atua Government Hospital in the Eastern Region of Ghana, serving a predominantly semi-urban and rural population from diverse socioeconomic backgrounds. The hospital provides general medical services and specialised HIV care through its antiretroviral therapy (ART) clinic, which currently offers medication dispensing, clinical monitoring, adherence counselling, and limited psychosocial support to approximately 200 ALHIV. The facility was selected due to its established adolescent HIV care programme and the willingness of hospital administration to support research aimed at improving mental health services for young people living with HIV.

### Study design

This study employed a qualitative descriptive design and constitutes a distinct, analytically independent component of the culturally adapted MFGT trial. All data were collected during the baseline assessment period, before any participant had received any component of the intervention, ensuring that findings reflect adolescents’ pre-intervention psychological realities rather than treatment effects. The qualitative and quantitative components of the trial address fundamentally different questions and are therefore analytically separate, with this study focusing exclusively on the subjective experiences, appraisal processes, and coping strategies of ALHIV prior to structured support.

### Study participants and sampling

Participants were adolescents receiving HIV care at Atua Government Hospital, selected via purposive maximum-variation sampling to ensure heterogeneity in age, gender, duration of HIV status awareness, and family structure, thereby capturing a broad range of psychological and social experiences rather than a narrowly defined subgroup. Eligibility criteria included confirmed HIV diagnosis, status awareness for at least 6 months, current ART enrolment, ability to communicate in English, Dangbe, or Twi, and informed assent with caregiver consent for those under 18 years. The six-month threshold is anchored to status awareness (the point at which the adolescent was told of, or came to know, their HIV status) rather than to date of biological diagnosis; because most participants in this cohort acquired HIV perinatally, diagnosis often occurred in infancy, years before disclosure to the child. Status awareness was therefore the psychologically meaningful reference point for this study, and the six-month threshold was set to allow sufficient time for adolescents to move beyond acute, immediate post-disclosure reactions and to have begun forming the appraisals and coping responses that are this study’s analytic focus (24), consistent with the trial’s baseline assessment window. The initial target range was 10 to 19 years, consistent with the WHO definition of adolescence. However, because many perinatally infected adolescents in this setting are not disclosed to until later in adolescence, a substantial proportion of eligible young people receiving HIV care at the facility had not yet reached the six-month status-awareness threshold within this age band. To avoid systematically excluding the experiences of late-disclosed adolescents, a population of particular relevance to a family-based intervention targeting disclosure-related distress, the sample was extended to include two participants aged 21 and 22 years who had been disclosed to only recently. “Developmental and social embeddedness” within the adolescent cohort was assessed against four explicit criteria: (a) residence with a parent or guardian rather than independently, (b) enrolment in secondary or post-secondary education rather than full-time employment, (c) financial dependence on family, and (d) unmarried status without children that is, neither participant had yet taken on the social or legal roles that typically differentiate young adulthood from adolescence in this context; both met all four criteria. This extension falls within the WHO’s broader 10 to 24 year “young people” category and follows precedent in comparable qualitative HIV research that extends recruitment beyond age 19 to preserve developmental and experiential continuity. During analysis, the research team compared the codes generated from these two participants’ transcripts against those generated from the remainder of the sample. Their accounts contributed to the same subthemes as younger participants (including family-based stigma, fatalistic reasoning, and religious coping), and no coder or team member flagged their data as qualitatively distinct during coding or theme development; no subtheme was unique to, or absent from, their transcripts. We note this as a qualitative, team-judgement comparison conducted during the coding process rather than a formal analytic technique, and we report it with that caveat rather than as confirmation that no age-related difference exists. This is nonetheless acknowledged as a limitation, since the transferability of findings to a strictly defined 10 to 19 year population cannot be assumed. We considered presenting these two participants as a separate sub-analysis or excluding them to preserve a narrower age-defined sample; we opted instead to retain them, integrated within the main sample, because separating them would imply a stronger claim of two-group difference than our comparative check supports, while excluding them would remove precisely the late-disclosure experiences this extension was designed to capture. Healthcare providers assisted in identifying eligible participants but were not involved in recruitment to minimise coercion. Sample adequacy was judged using the concept of information power (27) rather than a purely numeric saturation target: because the study aim was narrowly defined (psychological appraisal, stigma, and coping among ALHIV in one district), the purposive maximum-variation sample was information-rich relative to that aim, dialogue quality was supported by experienced facilitators and a conversational, break-permitting interview style, and analysis used an established, theory-informed coding framework, together supporting adequate information power despite the modest sample size. Consistent with this, after each data collection encounter, the first author and a research assistant reviewed the transcript and coding memos jointly to establish whether new codes or thematic content had emerged. Recruitment continued until two consecutive encounters (the second focus group discussion and the third in-depth interview) yielded no new codes; we treat this as evidence that saturation was reached for the specific phenomena explored in this sample and context, rather than as a claim that the full range of possible ALHIV experience had been captured. This determination was made jointly by the first author and the research assistants and confirmed in discussion with the supervisory team, yielding a final sample of 12 participants.

### Design of instruments

Data were collected through focus group discussions (FGDs) and in-depth interviews (IDIs), a methodological triangulation selected to capitalise on the complementary strengths of each approach: FGDs elicited group-level norms and shared understandings, whilst IDIs allowed more sensitive, individually specific material to surface (28). Rather than being analysed as two separate datasets and merged only at the reporting stage, FGD and IDI transcripts were coded together within a single, unified coding framework, applied in the chronological order in which data were collected. Codes generated from the FGDs were subsequently tested against, and refined using, the IDI transcripts, and vice versa, so that individual-level nuance from the interviews could elaborate and, where relevant, qualify group-level patterns identified in the focus groups. This integrated approach is reflected in the final thematic structure, in which no theme or subtheme derives exclusively from one data source. The two sources nonetheless elicited distinguishable kinds of material, consistent with their different purposes: FGDs tended to surface more normative, socially shared framing (for example, generalised statements about how community members respond to ALHIV), whereas IDIs elicited more personal and occasionally more sensitive disclosures, including suicidal ideation and explicit accounts of religious condemnation, which participants were less likely to voice in a group setting. Religious stigma and suicidal ideation were more prominently and specifically articulated in the IDI data, while community-level surveillance and peer exclusion were more richly described in the FGDs, where participants could corroborate and build on one another’s accounts. We treat this difference as expected from, rather than a threat to, the rationale for combining the two methods (28): each method surfaced the register of data it is best suited to elicit, and the integrated coding approach was designed to capture both registers rather than to treat them as interchangeable.

Two complementary instruments were developed through a literature review and expert consultation in adolescent HIV care and mental health. Both were theoretically anchored in Lazarus and Folkman's (24) Stress and Coping Theory and Link and Phelan's (25) stigma framework, which guided domain identification: (1) initial reactions to and understanding of diagnosis, (2) ongoing emotional and psychological experiences, (3) experiences of stigma across family, religious, and community contexts, (4) social and educational functioning, and (5) coping strategies and sources of support. Theoretical constructs were operationalised into accessible language for adolescents rather than posed as abstract concepts: secondary appraisal, for example, was translated into concrete questions about who participants could turn to and what happened when they tried, and Link and Phelan’s stigma components were operationalised through questions about specific instances of being treated differently, spoken about, or excluded, rather than through direct reference to “labelling” or “status loss.” The FGD guide used open-ended questions to elicit group-level perspectives, whilst the semi-structured IDI guide explored more personal dimensions including emotional responses and coping. Both were pilot-tested with two eligible adolescents not included in the final sample, allowing for refinement prior to data collection; piloting led to simplification of academically phrased items and to resequencing so that more sensitive questions (religious stigma, suicidal ideation) were placed later in the guide, after rapport had been established. The interview guides are provided as Supplementary material.

### Data collection procedure

Data collection occurred between February and March 2025, following the Consolidated Criteria for Reporting Qualitative Research (COREQ) guidelines (29) to ensure comprehensive reporting.

### Focus group discussions

Two FGDs were conducted at Atua Government Hospital: the first with four ALHIV (3 females, 1 male) and the second with five ALHIV (4 females, 1 male), composed to ensure age variation whilst maintaining developmental proximity. Each lasted 90 to 120 min, facilitated by the first author, a PhD candidate with expertise in qualitative research, adolescent HIV, and mental health, supported by a research assistant serving as translator and note-taker. Participants contributed in English, Twi, or Dangbe with real-time translation provided. Ground rules emphasising confidentiality, respect, and the right to withdraw were established collectively, and all sessions were audio-recorded with permission.

### In-depth interviews

Three ALHIV were selected for IDIs based on unique experiences, ability to articulate complex emotional processes, and variation in family structure, duration since diagnosis, and psychological challenge manifestation. IDIs were conducted online via telephone and Google Meet in English, facilitated by the last author, and lasted 60 to 90 min each. A conversational approach progressed from general to sensitive topics, with active listening practised throughout and breaks offered during emotional moments.

Field notes were recorded during and immediately after all sessions. Transcripts were produced verbatim; Twi and Dangbe segments were translated by two independent translators and verified against audio recordings prior to analysis.

### Data analysis

Data were analysed using Braun and Clarke’s (2017) six-step reflexive thematic analysis, selected for its adaptability in exploring complex, sensitive health experiences. Analysis was theoretically informed by Lazarus and Folkman's (24) Stress and Coping Theory, anchoring interpretation of how adolescents appraised HIV-related stressors and perceived coping resource availability, and Link and Phelan's (25) stigma framework, guiding attention to labelling, stereotyping, cognitive separation, status loss, and discrimination across family, religious, and community contexts. Both frameworks sensitised the analysis without constraining inductive theme generation; concretely, this sensitisation shaped the initial coding architecture in QDA Miner, in which appraisal-related codes were grouped under parent nodes for primary and secondary appraisal, and stigma-related codes were grouped under parent nodes corresponding to Link and Phelan’s five components, with inductive sub-codes nested beneath these theoretically derived parent categories rather than generated in a purely data-driven hierarchy.

The first author and two research assistants familiarised themselves with all transcripts, recording memos on emotional intensity, recurring concerns, and contrasting narratives. The first author and one research assistant independently open-coded the first four transcripts, generating an initial 147 codes, including “fear of death” and “medication avoidance.” These independent codes were then compared line by line; areas of disagreement, most often differences in the level of abstraction rather than substantive interpretation, were resolved through discussion, with the second author consulted as adjudicator where consensus could not be reached. Because reflexive thematic analysis treats coding as an interpretive rather than a purely procedural exercise, no numerical intercoder agreement statistic was calculated; instead, credibility was pursued through this structured discussion process and through the researcher triangulation and audit trail described below (30). The calibrated coding framework was then applied systematically by the first author across the full dataset using QDA Miner software, with a random 20% of transcripts independently re-coded by the second research assistant to check consistency of application. Seven preliminary themes were identified and subsequently collapsed or expanded; “educational concerns” was merged into “social isolation” as it stemmed from stigma-related school avoidance, consistent with Link and Phelan’s concept of cascading status loss. An audit trail and independent transcript review enhanced rigour. Reporting follows the Consolidated Criteria for Reporting Qualitative Research (COREQ) (29) throughout; where frequency descriptors (for example, “several” or “some”) are used in the Results, these reflect a qualitative impression of relative prevalence within the coded dataset rather than precise counts, consistent with the interpretive orientation of reflexive thematic analysis.

The final thematic structure comprised five core themes: Emotional and Mental Distress, Stigma and Discrimination, Social Isolation, Treatment Adherence Challenges, and Coping Mechanisms, each supported by nuanced subthemes.

### Rigor and reflexivity

Several strategies were employed to enhance methodological rigor. Credibility was strengthened through prolonged engagement with participants, member checking (in which preliminary findings were shared with a subset of participants for feedback), and peer debriefing within the research team. Transferability was addressed through rich, thick description of the context and participants. Dependability was ensured through maintenance of an audit trail documenting all methodological decisions. Confirmability was supported through researcher triangulation and reflexive journaling.

Reflexivity was a central consideration throughout the research process. The research team acknowledged their positionality as healthcare researchers who were not living with HIV and reflected on how this might influence data collection and interpretation. On disclosure, the team recognised that its clinical/healthcare affiliation may have made some participants reluctant to disclose non-adherence or negative feelings about care, for fear this could affect their treatment relationship, despite assurances of confidentiality and no effect on healthcare; this is flagged explicitly here as a plausible source of under-reporting and is connected to the social-desirability-bias limitation discussed below. On the team’s HIV-negative status, the researchers did not claim or imply shared experiential understanding with participants during data collection, relying instead on active listening, non-leading questions, and, where relevant, explicit acknowledgement that the interviewer could not know first-hand what the participant was describing, a deliberate stance intended to avoid presuming shared understanding across this status difference. The first author’s clinical background in mental health nursing and prior professional exposure to adolescent HIV care shaped an initial sensitivity to psychological distress and coping language, which was treated in reflexive memos as an analytic lens to be made explicit rather than bracketed out; for example, an early tendency to code emotional accounts directly into clinical categories such as “depression” was identified during team debriefing and deliberately revised so that Results retained participants’ own descriptive language, with clinical or theoretical framing reserved for the Discussion. Regular reflexive discussions among the research team addressed these and other potential biases, preconceptions, and power dynamics, including the adult-researcher/adolescent-participant power differential inherent in the interview and facilitation roles; in practice, this differential was managed by allowing participants to choose between a first name or a numerical identifier, conducting sessions away from clinical staff and in private, permitting breaks and withdrawal at any point without requiring justification, and periodically checking that questions were being understood as intended rather than assuming comprehension. The primary interviewer, a Ghanaian with experience working with ALHIV, maintained a reflexive journal documenting personal reactions, challenges, and evolving understandings throughout the data collection process, which was reviewed periodically by the wider team to surface assumptions that might otherwise have gone unexamined.

### Ethics consideration

Ethical approval was obtained from the Ghana Health Service Ethics Review Committee (GHS-ERC: 004/07/24), with institutional permission granted by the Lower Manya Krobo Municipal Health Directorate and the administration of Atua Government Hospital. The study adhered to the Declaration of Helsinki and ethical principles for research with vulnerable adolescents. Protective measures included informed consent and assent in participants’ preferred language, guardian consent for those under 18 years, confidentiality through numerical identifiers and secure data storage, voluntary participation with no effect on healthcare, access to a clinical psychologist, and modest transportation reimbursement (100 to 200 Ghana Cedis). Team members received training in ethical conduct of adolescent HIV research.

### Findings

#### Sociodemographic characteristics

Table 1 summarises the sociodemographic characteristics of 12 adolescent participants, mostly female (83.3%), with a mean age of approximately 16.4 years (range 10–22 years); most participants (58.3%) fell within the 15 to 19 year band. The majority were students (83.3%), primarily at the SHS and JHS levels. About 41.6% had known their HIV status for 1–5 years. Most lived with grandparents (33.3%) or a single parent, and guardian marital statuses were mixed with majority being married (33.3%).

**Table 1:** Sociodemographic characteristics of ALHIV.

Variable	Category	Frequency	Percentage (%)
Age	10–14	3	25
	15–19	7	58.3
	20–22	2	16.7
Gender	Male	2	16.7
	Female	10	83.3
Educational level	Primary	1	8.3
	Junior High School	4	33.3
	Senior High School	5	41.6
	Completed JHS	2	16.7
Occupation	Student	10	83.3
	Learning a trade	2	16.7
Years since HIV diagnosis awareness	<1 year	3	25
	1–5 years	5	41.6
	>5 years	4	33.3
Lives with	Parents	2	16.7
	Mother only	3	25
	Grandparents	4	33.3
	Siblings	3	25
Guardian’s marital status	Single	3	25
	Married	4	33.3
	Widowed	3	25
	Divorced	2	16.7

### Main findings

#### Themes and subthemes

This comprehensive thematic analysis explores the complex psychological landscape of adolescents living with HIV/AIDS, examining their emotional distress, experiences of stigma, social isolation, treatment challenges, and coping strategies (Table 2).

**Table 2:** Themes and subthemes with code descriptions.

Theme	Subtheme	Code	Code description	Representative quote
T1 emotional and mental distress	1.1 Suicidal ideation	SUI-01	Post-diagnosis suicidal ideation	“. I even wanted to kill myself because I could not believe I have the HIV. — ALHIV-1”
		SUI-02	Fatalistic thinking undermining ART adherence	“. I will be feeling like not drinking the medicine because by all means I’ll die. — ALHIV-6”
	1.2 Fear of death	FEAR-01	Mortality anxiety from peer misinformation	“.if you drink the medicine, you’ll be good, but still you will die. — ALHIV-9”
		FEAR-02	Existential dread fuelling treatment avoidance	“.at times if I’m there, I will be feeling like not drinking the medicine… — ALHIV-4, ALHIV-6”
	1.3 Depressive tendencies	DEP-01	Episodic tearfulness triggered by HIV status awareness	“Anytime I sit down to think about the fact that I have the disease, I will start crying. — ALHIV-5”
		DEP-02	Anhedonia and withdrawal from school and peers	“Since I found out about the disease, I do not even like going to school anymore… — ALHIV-8”
T2 stigma and discrimination	2.1 Family-based stigma	FSTIG-01	Scapegoating — blamed for family misfortunes	“They say I’m the reason why my aunty is not married… — ALHIV-9”
		FSTIG-02	Geographic exclusion from the family home	“She even sent me to the village… where none of my family members were there… — ALHIV-9”
	2.2 Religious stigma	RSTIG-01	HIV reframed as witchcraft / spiritual contamination	“.the pastor said… someone gave it to me so I’m a witch. — ALHIV-8”
		RSTIG-02	Ostracism by religious community	Implicit in accounts of religious leaders’ condemnation — ALHIV-8
	2.3 Community & Peer stigma	CSTIG-01	Unauthorised status disclosure by family members	“My sister… went to tell her friends about my HIV status… — ALHIV-7”
		CSTIG-02	Subtle exclusion — silence, laughter, surveillance	“.if they are talking and they see that I’m coming, they just keep quiet… — ALHIV-11”
T3 social isolation	3.1 School avoidance	SCHAV-01	Deliberate absenteeism driven by disclosure fears	“Since last week I did not even go to school because of how my sister has been reacting… — ALHIV-2”
	3.2 Restricted social interactions	RSOC-01	Self-imposed withdrawal to avoid gossip and stigma	“I do not play with them [peers]. I am trying to avoid gossips and name calling. — ALHIV-9”
	3.3 Limited support systems	SUPP-01	Complete absence of a trusted confidant	“Participants responded ‘NO’ when asked if anyone was available to talk to — Multiple”
		SUPP-02	Geographically contingent / conditional support	“If I’m sad and my auntie is not around, I do not have anybody to talk to… — ALHIV-11”
		SUPP-03	Fully internalised coping — isolated, secretive distress	“I just stay in the room and lock myself and pray so that no one can hear me. — ALHIV-3”
T4 treatment adherence challenges	4.1 Negative attitude toward medication	ADH-01	ART aversion embedded in existential / fatalistic reasoning	“Yes oo, I do not like them [ARVs]… by all means I’ll die. — ALHIV-4”
	4.2 Lack of treatment support	ADH-02	Caregiver refusal to facilitate clinic attendance	“.they’ll say they are tired. Without them, it is difficult to go to the hospital… — ALHIV-6”
T5 coping mechanisms	5.1 Religious coping	COPE-01	Prayer and scripture as comfort and resilience resource	“.the bible because my class teacher has been coming to me to read the bible to me. — ALHIV-11”
	5.2 Family connection	COPE-02	Symbolic connection to family (including deceased) for emotional anchoring	“I always take my mum’s picture. My mum is no more. I will be watching her picture everyday… — ALHIV-8”
	5.3 Personal resilience	COPE-03	Self-aware emotional intelligence as a coping capacity	“I am a type of person who is emotionally intelligent and have personal resilience. — ALHIV-10”
		COPE-04	Active self-encouragement; deliberate independence	“.sometimes you try to encourage yourself. You cannot always depend on others. — ALHIV-10”

### Theme 1: Emotional and mental distress

#### Subtheme 1.1: suicidal ideation

Suicidal ideation was identified as a significant subtheme within the broader category of emotional and mental distress experienced by adolescents living with HIV/AIDS. This manifestation of severe psychological distress appears to be triggered by specific events or realizations in the HIV journey. Several participants described suicidal ideation occurring shortly after being discharged from the hospital:

“When I was discharged, I went home... In the night when everybody was asleep, I even wanted to kill myself because I couldn’t belief I have the HIV.” *(ALHIV 1)*

The presence of suicidal ideation underscores the need for comprehensive mental health screening and support as integral components of HIV care for adolescents. The quotes suggest that healthcare providers should be particularly vigilant during the post-diagnosis period and attentive to signs of treatment fatigue, as these appear to be high-risk periods for suicidal thoughts among adolescents living with HIV/AIDS.

This pattern is consistent with catastrophic primary appraisal (24): HIV is evaluated in the moment of realisation not as a manageable condition but as an irreversible existential threat, a framing discussed further below.

#### Subtheme 1.2: fear of death

Fear of death was a further significant subtheme within the emotional and mental distress experienced by adolescents living with HIV/AIDS. This fear manifests in various ways, from explicit concerns about mortality to fatalistic thinking that influences treatment adherence. Adolescents’ understanding of HIV and its relationship to death appears heavily influenced by various information sources, often with unintended negative consequences. One participant recounted seeking information from peers, revealing how inaccurate or incomplete information can heighten fear of death.

“When I was in Accra, we read about HIV then after closing I asked a friend that can anyone who is having HIV/AIDS be alive for so many years? What she told me was, if you drink the medicine, you’ll be good, but still you will die.” *(ALHIV 9)*

Perhaps most concerning is how fear of death can paradoxically lead to treatment avoidance or non-adherence:

“So, at times if I’m there, I will be feeling like not drinking the medicine because by all means I’ll die.” *(ALHIV 6)*

This quote reveals a dangerous cycle: the belief in inevitable death despite treatment leads to questioning the purpose of medication adherence. The participant expresses a fatalistic logic—if death is certain regardless of treatment, then the burden of daily medication seems pointless. This thinking pattern demonstrates how fear of death can directly undermine the very behaviors that would extend life and health.

This fatalistic reasoning illustrates a secondary appraisal in which coping resources, in this case, the perceived efficacy of treatment itself, are judged as unavailable or futile (24); we return to its implications for adherence in the Discussion, alongside other plausible contributors to non-adherence.

#### Subtheme 1.3: depressive tendencies

Sadness and depressive symptoms were described as pervasive emotional experiences among adolescents living with HIV/AIDS. These manifestations range from transient episodes of tearfulness to more persistent patterns of low mood and withdrawal, affecting multiple domains of the adolescents’ lives; no clinical diagnosis was made as part of this study, and these are reported as participants’ subjective depressive experiences rather than confirmed clinical depression. Crying appeared as a common expression of emotional distress, often triggered by thoughts about their HIV status:

“Anytime I sit down to think about the fact that I have the disease, I will start crying.” *(ALHIV 5)*

Beyond episodic crying, participants described symptoms consistent with more pervasive depression, including loss of interest in previously enjoyable activities:

“Since I found out about the disease, I don’t even like going to school anymore. And I don’t really talk to my friends, because my friends will get some.” *(ALHIV 8)*

This withdrawal from education and peer relationships reflects a loss of interest in previously valued activities alongside growing social isolation. The experiences of these adolescents underscore that such depressive experiences represent not merely transient emotional reactions but significant challenges to overall well-being and development, warranting attention and structured psychosocial support (see Discussion).

Consistent with the Stress and Coping framework, this withdrawal can also be read as a secondary-appraisal consequence: as valued sources of support and activity recede, the coping resources available to the adolescent narrow further, compounding distress.

### Theme 2: Stigma and discrimination

#### Subtheme 2.1: family-based stigma

Family-based stigma was a particularly painful dimension of the stigma experience for adolescents living with HIV/AIDS. This form of rejection from those expected to provide unconditional support creates unique psychological wounds, with profound implications for adolescents’ sense of belonging, self-worth, and emotional security. One of the most damaging forms of family-based stigma involves being blamed for family misfortunes that have no logical connection to the adolescent’s HIV status:

“They say I’m the reason why my aunty is not married. She even sent me to the village… where none of my family members were there and my brothers came for me.” *(cries) (ALHIV, 9)*

Family-based stigma represents a particularly pernicious form of rejection for adolescents with HIV because it undermines the very foundation of security and belonging necessary for healthy development. Interventions should address not only the adolescent’s coping skills but also family systems, and cultural beliefs that enable and authorize such rejection within supposedly protective relationships.

This account illustrates Link & Phelan’s status loss enacted spatially rather than only relationally: being sent away severs the adolescent from the family home that is expected to anchor belonging, and does so through physical relocation rather than only through words or attitudes. The psychological weight of this experience operates on at least three levels: the rupture of belonging, as the adolescent is displaced from the very relationships meant to provide security; the geography of rejection, in which exile to “the village” makes the rejection materially visible and difficult to deny or reframe; and the practical consequence of losing proximity to established sources of support, schooling, and routine. Read together, these dimensions suggest that family-based stigma of this kind may be experienced not as a single act of rejection but as a compounding loss across emotional, spatial, and practical domains simultaneously.

#### Subtheme 2.2: religious stigma

Religious stigma was a particularly destructive form of stigma experienced by adolescents living with HIV/AIDS. This form of stigma carries unique power due to its perceived divine authority and its ability to transform a medical condition into a moral or spiritual failing, with profound implications for an adolescent’s identity development and social acceptance.

A central feature of religious stigma involves reframing HIV as evidence of spiritual contamination rather than a medical condition:

“A pastor came to our house when I was in the hospital... They said the pastor said that for me the disease, someone gave it to me so I’m a witch.” *(ALHIV 8)*

This quote reveals how religious figures reinterpret HIV through a supernatural lens, transforming its etiology from biomedical (virus transmission) to mystical (witchcraft).

In Link and Phelan's (25) terms, this reinterpretation functions as a distinctive act of labelling and stereotyping, one that additionally invokes status loss by recasting the adolescent as a moral or spiritual agent of harm (a “witch”) rather than a patient; we discuss below why this channelling through perceived divine authority makes the label unusually resistant to challenge.

#### Subtheme 2.3: community and peer stigma

Community and peer stigma also represents a particularly painful dimension of the stigma experience for ALHIV. This form of social rejection occurs at a developmental stage when peer acceptance is critically important for identity formation and psychological well-being, creating unique challenges for adolescents trying to navigate normal social development while managing a stigmatized health condition. A fundamental aspect of community stigma begins with unauthorized disclosure of HIV status:

“My sister... She too she likes talking to plenty people, so she went to tell her friends about my HIV status so any time I’m passing, they (community members who are mostly the adolescents peers) have been watching me.” *(ALHIV 7)*

This quote reveals how family members may breach confidentiality, initiating the spread of stigma through social networks and captures the sensation of being under constant surveillance, where normal activities like walking through public spaces become experiences of intense self-consciousness.

Peer stigma often manifests through subtle social signals rather than explicit rejection:

“Because sometimes, if they are talking and they see that I’m coming, they just keep quiet and just be laughing.” *(ALHIV 11)*

Community and peer stigma represents a particularly challenging dimension of the HIV experience for adolescents precisely because it occurs during a developmental period when social belonging is crucial for identity formation. The resulting isolation can create cascading negative effects across educational, psychological, and health domains, highlighting the importance of interventions that specifically address the social ecology surrounding adolescents with HIV/AIDS.

The surveillance and abrupt silence described here exemplify link and Phelan’s cognitive separation, an us-versus-them boundary enacted through everyday social micro-behaviours rather than explicit confrontation, which may be harder for adolescents to name, challenge, or seek support around than overt discrimination.

### Theme 3: Social isolation

#### Subtheme 3.1: school avoidance

School avoidance was a significant behavioral manifestation of the emotional and social challenges faced by adolescents living with HIV/AIDS. This pattern of educational disengagement represents not merely a temporary emotional response but a potentially life-altering consequence with implications for future opportunities and development. Concerns about privacy and unauthorized disclosure drive school avoidance:

“Since last week I didn’t even go to school because of how my sister has been reacting. She likes telling people about my sickness (HIV).” *(ALHIV 2)*

This statement reveals how vulnerability to unwanted disclosure creates school environments that feel unsafe for adolescents with HIV. The fear that others will learn about their status transforms school from a place of learning into a site of potential exposure and humiliation.

Avoidance here functions as emotion-focused coping directed at managing anticipated exposure rather than the underlying stressor (24); it is a secondary-appraisal response to an environment judged unsafe, even as it carries longer-term costs to educational participation.

#### Subtheme 3.2: restricted social interactions

Restricted social interactions were a significant pattern among ALHIV, reflecting both externally imposed limitations and self-protective withdrawal. This social restriction occurs during a developmental period when peer relationships are crucial for identity formation, social skill development, and emotional well-being. The most extreme form of social restriction involves total withdrawal from peer interactions:

“I don’t play with them [peers]. I am trying to avoid gossips and name calling.” *(ALHIV 9)*

This statement reveals social withdrawal as a conscious protective strategy. The adolescent makes a cost–benefit calculation, determining that the emotional safety gained through isolation outweighs the benefits of social connection.

This is a further instance of emotion-focused coping through avoidance; it also illustrates how secondary appraisal, here, a judgement that peer relationships offer more exposure to stigma than support, can rationally lead to a coping choice that nonetheless narrows the adolescent’s available relational resources over time.

#### Subtheme 3.3: limited support system

The narratives of adolescents living with HIV/AIDS revealed a consistent pattern of severely limited support systems, characterized by isolation, lack of emotional confidants, and reliance on minimal or distant sources of support. The most extreme manifestation of limited support involves having no one to discuss emotional challenges with. When asked if they have anybody to talk to and share their deepest pains and fears with, some participants responded with a brave “NO.”

Some narratives reveal that family support, when available, is highly conditional or inconsistent:

“If I’m sad and my auntie is not around, I don’t have anybody to talk to, but if I go to my mum’s place and I’m sad, she really encourages me a lot.” *(ALHIV 11)*

This statement from a participant illustrates how support may be geographically contingent and inconsistently available—present in one household but absent in another, or dependent on specific family members being physically present.

Limited support places the entire burden of emotional processing on the adolescent:

“I just stay in the room and lock myself and pray so that no one can hear me.” *(ALHIV 3)*

This description reveals a completely internalized emotional coping system, where difficult feelings must be processed entirely within the adolescent’s own psychological resources without the benefit of co-regulation or external perspective that supportive relationships would provide.

Taken together, these accounts describe secondary appraisal under conditions of severely depleted external coping resources: with few or no external supports judged available, coping is forced inward, a pattern discussed further below alongside its implications for intervention design.

### Theme 4: Treatment adherence challenges

#### Subtheme 4.1: negative attitude toward medication

Negative attitudes toward antiretroviral medication were a significant challenge faced by adolescents living with HIV/AIDS. These attitudes ranged from simple dislike to more complex existential reasoning that undermined adherence motivation, with potential serious implications for health outcomes and disease management. The most straightforward manifestation of negative medication attitudes is simply expressed dislike:

“Yes oo, I don’t like them.” *(referring to Antiretroviral drugs) (ALHIV 4)*

This statement reveals a straightforward but consequential resistance to daily medication. Medication requirements may conflict with adolescents’ developmental drive for autonomy and self-determination, creating tension that manifests as expressed dislike or rejection; as reported elsewhere in the data (see Subtheme 1.2), this dislike was, for some participants, further compounded by fatalistic beliefs about the futility of treatment. We interpret this fatalism-linked pattern, cautiously, as one plausible coping-related contributor to reduced adherence motivation for the subset of participants who expressed it, rather than as a general reframing of non-adherence in this sample. Participants did not themselves describe their medication-taking behaviour using the language of coping; the coping interpretation is our analytic reading of the appraisal logic evident in their words, not a self-description they offered. Other, non-mutually-exclusive explanations for non-adherence are equally plausible and are documented elsewhere in the data, including the practical access barriers described in Subtheme 4.2; we do not have evidence to adjudicate between these explanations and present the coping interpretation as one contributor among several rather than as the primary or confirmed mechanism.

#### Subtheme 4.2: lack of treatment support

Lack of treatment support was identified as a significant challenge for adolescents living with HIV/AIDS. This absence of practical assistance with medical care represents a critical gap in the support system necessary for effective disease management, with potentially serious implications for treatment adherence and health outcomes. The most concrete manifestation of inadequate treatment support is the denial of transport to medical appointments:

“Sometimes, if I say they should come and escort me to the hospital to collect my drugs, they’ll say they are tired. Without them, it is difficult to go to the hospital on clinic days.” *(ALHIV 6)*

This statement reveals a fundamental barrier to accessing medical care—the simple inability to physically reach treatment facilities without assistance. The adolescent’s need to request escort suggests they lack independent transportation means, creating dependency on others for accessing life-sustaining medication. The reported response also indicates that this essential support is inconsistently provided, subject to the convenience and willingness of family members rather than prioritized as a health necessity.

### Theme 5: Coping mechanisms

#### Subtheme 5.1: religious coping

Religious coping emerged as a significant strategy employed by adolescents living with HIV/AIDS to manage the psychological and emotional challenges of their condition. This approach to coping draws on spiritual resources, religious practices, and faith-based social support to provide meaning, comfort, and resilience in the face of significant life challenges. One manifestation of religious coping involves engagement with religious texts as a source of guidance and comfort:

“…And the bible because my class teacher has been coming to me to read the bible to me.” *(ALHIV 11)*

This statement reveals how scripture serves as a spiritual resource accessed both independently and through supportive relationships. Interventions can leverage religious coping as an important psychological resource while minimizing potential harms when they simultaneously support positive spiritual frameworks and address harmful religious interpretations of HIV.

This is an emotion-focused coping strategy in Lazarus and Folkman’s terms, directed at regulating distress through spiritual meaning-making rather than altering the stressor itself; its co-occurrence with religious stigma (Subtheme 2.2) within the same institutional domain is addressed as a distinct analytic point in the Discussion.

#### Subtheme 5.2: family connection

Family connection emerged as a crucial coping mechanism for adolescents living with HIV/AIDS, though these connections varied significantly in form, availability, and quality. These familial relationships provide emotional anchoring, identity continuity, and practical support, even when limited or primarily symbolic in nature. In the absence of living supportive family members, some adolescents maintain symbolic connections with deceased relatives:

“I always take my mum’s picture. My mum is no more. I will be watching her picture everyday if I am sad. It makes me happy.” *(ALHIV 8)*

This poignant description reveals how tangible representations (photographs) serve as emotional anchors and connections to lost supportive relationships. The ritualistic aspect of this practice (“everyday if I am sad”) suggests this has become a structured coping strategy that the adolescent intentionally employs during emotional distress.

This meaning-focused coping by Lazarus and Folkman, sustains a sense of connection and identity continuity independent of whether supportive family members are living, a variation on family connection discussed further below alongside the co-occurring finding that family can also be a source of stigma (Subtheme 2.1).

#### Subtheme 5.3: personal resilience

Personal resilience emerged as a significant internal resource that some adolescents with HIV/AIDS develop and utilize to navigate the challenges of their condition. This capacity for psychological adaptation and self-support represents a powerful intrinsic strength that exists alongside, and sometimes in place of, external support systems.

Some adolescents demonstrate remarkable self-awareness about their psychological capabilities:

“I am a type of person who is emotionally intelligent and have personal resilience.” *(ALHIV 10)*

This statement demonstrates sophisticated self-understanding, with the adolescent not only possessing resilience but being consciously aware of this quality.

Resilience manifests through deliberate self-supportive behaviors:

“...sometimes you try to encourage yourself. You can’t always depend on others.” *(ALHIV 10)*

This description reveals resilience as an active process rather than merely a passive characteristic. This also represents a form of internal psychological resourcefulness—the ability to be both the provider and recipient of encouragement.

This pattern illustrates Lazarus and Folkman's (24) problem-focused coping directed inward: rather than modifying the external stressor, the adolescent actively regulates their own emotional state through deliberate self-directed strategies, a micro-level self-regulatory process discussed further in relation to intervention design below.

## Discussion

This study explored the mental and emotional challenges experienced by ALHIV in the Lower Manya Krobo District of Ghana, interpreted through Lazarus and Folkman's (24) Stress and Coping Theory and Link and Phelan's (25) stigma framework. The data reveal a coherent pattern: adolescents encounter HIV as an overwhelming primary stressor, perceive their coping resources as severely depleted by multi-dimensional stigma and social isolation, and consequently resort to both adaptive and maladaptive coping strategies in the face of inadequate systemic support.

The severe psychological distress documented, including suicidal ideation, fear of death, and pervasive depressive symptoms, reflects catastrophic primary appraisal whereby HIV diagnosis is evaluated not as a manageable condition but as an irreversible existential harm (24). This aligns with prior evidence of elevated mental health burden among ALHIV (9, 16, 31), but the qualitative approach here identifies specific high-risk windows, notably the post-diagnosis period and treatment fatigue episodes, not adequately captured by quantitative studies. Fatalistic reasoning about inevitable death, extending Mutumba et al. (15), represents avoidant emotion-focused coping that paradoxically undermines adherence. Depressive symptoms were compounded by hostile secondary appraisal: when adolescents perceived their social environments as threatening and support as absent, adaptive coping capacity collapsed (8). Interventions should therefore address cognitive appraisal and the social ecology of coping, not individual symptom management alone.

Three distinct stigma forms were characterised, each operating through different Link and Phelan (25) mechanisms and depleting coping resources in distinct ways. Family-based stigma is theoretically the most damaging because it destroys the primary coping buffer secondary appraisal depends upon most; when families enact labelling, stereotyping, and discrimination through blame and physical exclusion, the adolescent’s entire proximal support network collapses (32, 33). Religious stigma emerged as a particularly striking empirical pattern: by reframing HIV aetiology as witchcraft through perceived divine authority, it appears to operate through the same core mechanisms Link and Phelan (25) describe, labelling, stereotyping, and status loss, but channelled through a culturally specific pathway, namely perceived divine authority, that may make the label unusually resistant to challenge, since contesting it can mean contesting spiritual authority itself. We present this as evidence for the importance of attending to religious institutions as a distinct site through which existing stigma mechanisms operate, consistent with Gaskins et al. (34) and Medved Kendrick (20); establishing whether this pathway is best understood as a variant of existing mechanisms or as a genuinely distinct mechanism would require comparative data beyond the scope of this study. We retain religious stigma as an analytically distinct subtheme, rather than a sub-type of community or family stigma, for three reasons: its source of authority is categorically different from peer or familial authority, changing how contestable the label is to the adolescent; it operates through a specific etiological reframing (spiritual contamination or witchcraft) not present in the family- or community-stigma data; and, uniquely among the three stigma forms documented here, it also functions, for the same or different adolescents, as a coping resource (Subtheme 5.1), a paradox with no equivalent in family or community stigma that risks being obscured if the categories are merged (discussed further below). Community and peer stigma, enacted through subtle signals including surveillance and abrupt silence, sustains chronic threat appraisal; its initiation through unauthorised family disclosure highlights a pathway community-level campaigns alone cannot address (35, 36).

Social isolation, encompassing school avoidance, restricted peer interactions, and severely limited support systems, is best understood as both a consequence of depleted secondary appraisal resources and a self-perpetuating cycle further limiting them. School avoidance extends Orkin et al. (22) by documenting the specific appraisal process: school is experienced as a stigma-exposure site, making avoidance a rationally coherent though ultimately harmful coping response. Most concerning was the finding that some participants lacked any confidant, signalling near-total relational coping resource collapse more profound than quantitative support scales typically detect. Betancourt, Grelotti, and Hansen (14) identify social support as the central resilience mechanism in HIV-affected youth; for a substantial subset here, that mechanism had effectively ceased to function.

Non-adherence to antiretroviral therapy may, for the subset of participants who expressed fatalistic beliefs, be understood in part as a coping-related response to failed appraisal (24) rather than as a knowledge or access deficit alone; when primary appraisal frames treatment as futile and practical coping resources such as transport assistance are withheld, reduced adherence motivation becomes psychologically plausible. We present this as one contributor among several rather than as a general reframing of non-adherence in this sample: participants did not describe their own medication-taking behaviour in the language of coping, and our data cannot rule out, and indeed elsewhere document, other explanations including structural access barriers (Subtheme 4.2), alongside pill fatigue, side effects, and forgetfulness reported in the wider literature. Programmes addressing pill organisation or health literacy without also engaging appraisal processes are, on this reading, unlikely on their own to produce sustained change, extending Abubakar et al. (37).

The three coping mechanisms identified map onto Lazarus and Folkman's (24) taxonomy whilst revealing culturally significant extensions. Religious coping represents emotion-focused coping through prayer, scripture, and faith community engagement; its co-occurrence with religious stigma within the same institutional context constitutes a striking pattern in this dataset, one not anticipated by either framework and, to our knowledge, not previously described in the Ghanaian literature, though further research would be needed to establish how widespread it is (20, 38). Family connection, including comfort drawn from a deceased mother’s photograph, illustrates meaning-focused coping and extends Bhana et al. (13) by demonstrating that attachment representations sustain coping function even without living family members. Personal resilience, manifested through deliberate self-encouragement and verbalised emotional self-awareness, represents problem-focused coping directed inward, extending Betancourt, Grelotti, and Hansen (14) by specifying the micro-level self-regulatory processes through which some ALHIV maintain well-being despite severe adversity. Intervention models should explicitly cultivate these internal capacities.

### Variability across and within relational and institutional contexts

The themes reported above are not mutually exclusive, uniformly experienced categories, and a full account of the data requires attending explicitly to their overlap and tension rather than treating each as a separate, internally homogeneous group of participants. Most notably, family connection (Subtheme 5.2) and family-based stigma (Subtheme 2.1) coexisted in this dataset: some participants experienced family primarily as a stigma source, others primarily as a coping resource, and support was, for several participants, geographically or relationally contingent within the same family, present from one relative and absent from another (consistent with Subtheme 3.3). The same pattern recurred for religion: some participants experienced religious institutions primarily as a source of condemnation (Subtheme 2.2), others primarily as a source of comfort (Subtheme 5.1), and religious stigma and religious coping were, for at least one participant, both present within the same account. We treat this coexistence as a substantive finding in its own right rather than an inconsistency to be resolved: relational and institutional contexts in this population are rarely uniformly protective or uniformly harmful, which has direct implications for intervention design, discussed next.

### The religious stigma-coping paradox and its implications for intervention design

The finding that religious contexts functioned as both the most potent stigma source and the most widely accessed coping resource complicates a straightforward reading of “religious coping” in the wider HIV literature, where it is often treated as an unconditionally positive resource; here, the same institutional context could be simultaneously protective and harmful, at times for the same adolescent. This has practical implications for intervention design. First, individual pastoral or spiritual support, prayer, scripture engagement, and faith-based emotional comfort, can be preserved and even actively strengthened as a coping resource, while congregational teaching that frames HIV as spiritual contamination or divine punishment is a distinct and separable target for anti-stigma engagement; treating these as a single undifferentiated “religion” variable risks either withdrawing a valued coping resource or leaving harmful teaching unaddressed. Second, partnering with sympathetic religious leaders to deliver messaging that reframes HIV in medical rather than moral terms, while retaining the spiritual language of comfort and belonging that adolescents draw upon, may allow religious leaders to be engaged as allies without requiring wholesale theological change. Third, lay or peer support workers embedded within faith communities could model non-stigmatising responses to HIV in everyday congregational life, offering a lower-friction route to shifting norms than top-down doctrinal change. We offer these as directions for intervention design rather than as tested strategies, since this study was not designed to evaluate them.

### Implications and recommendations

The recommendations below are offered as forward-looking implications of these findings for intervention design generally, and for the MFGT trial of which this study forms the baseline qualitative component specifically; they are not presented as a retrospective account of curriculum decisions already made in that trial’s cultural adaptation.

This study’s findings suggest a multi-level approach to supporting adolescents with HIV/AIDS. At the clinical level, mental health screening should be integrated into HIV care, particularly during post-diagnosis and treatment fatigue periods, with standardized suicide risk assessment. Interventions should target family systems rather than just individuals, while engaging constructively with religious beliefs that can function as both coping resources and sources of stigma. At the policy level, educational accommodations should address both practical barriers and psychosocial concerns, while healthcare systems should improve treatment access through transportation support and flexible service delivery models. Anti-stigma initiatives should target religious, cultural, and social dimensions beyond medical misconceptions. Future research should develop culturally appropriate interventions, conduct longitudinal studies of psychological trajectories, investigate resilience mechanisms, and explore the complex intersection of religious beliefs and HIV stigma, particularly in contexts where traditional frameworks significantly influence illness interpretations.

These findings carry specific implications for nursing practice. Nurses and other frontline HIV care providers are typically the first point of contact following diagnosis and disclosure, placing them in a position to identify the high-risk post-diagnosis window described above and to conduct brief, routine psychological check-ins rather than relying solely on adolescents to self-report distress. Nurse-led adherence counselling could be strengthened by explicitly exploring the appraisal-based reasoning identified here, for example, gently eliciting and addressing beliefs that treatment is futile, rather than assuming non-adherence reflects forgetfulness or poor health literacy alone. Given the prominence of family-based and religious stigma in these findings, nurses and mental health practitioners are well placed to facilitate family psychoeducation sessions and to build collaborative relationships with local religious leaders, so that faith communities can be engaged as potential allies in reducing stigma rather than left as an unaddressed source of it. HIV counsellors could also incorporate structured screening for social isolation, including simple questions about whether the adolescent has anyone to confide in, as part of routine adherence counselling, given how starkly some participants described the absence of any confidant.

### Limitations of the study

Several limitations should be considered when interpreting these findings. Consistent with the aims of purposive qualitative inquiry, the relatively small sample size is not treated here as a limitation on statistical generalizability, which is not the standard against which this design should be judged; rather, readers should judge the transferability of these findings to their own settings based on the rich contextual description provided. Participants were recruited from a single healthcare facility in one district, potentially missing experiences of adolescents not engaged with care and limiting the transferability of findings to other settings, including urban contexts and other regions of Ghana with different cultural and religious profiles. Relatedly, although healthcare providers were not involved in recruitment decisions, their role in identifying potentially eligible participants may have introduced a selection bias toward adolescents who were more visibly engaged with, or in better standing with, the clinical team, potentially under-representing more disengaged or non-adherent adolescents. Participants’ awareness of the study’s association with a clinical trial evaluating a mental-health and adherence intervention may also have shaped their responses, for example by priming them to emphasise adherence-related distress. The cross-sectional nature of the study cannot capture how psychological experiences evolve. Additionally, most participants had perinatally acquired HIV, potentially limiting insights into the experiences of adolescents with behaviorally acquired HIV. As noted above, the inclusion of two participants aged 21 and 22 years, whilst justified methodologically, means the sample extends slightly beyond the WHO-defined adolescent age range, and findings should be interpreted with this in mind. Social desirability bias is also possible, particularly given that participants were recruited through, and interviewed in proximity to, their treatment facility, which may have shaped what they felt able to disclose about non-adherence or negative feelings towards care. The three in-depth interviews were conducted by telephone or video call rather than in person; whilst this format was chosen to protect confidentiality and reduce travel burden, it may have affected rapport-building and the emotional richness of the data compared with face-to-face interviews, and non-verbal cues could not be systematically captured. Finally, although Twi and Dangbe segments were translated by two independent translators and verified against audio recordings, some translation and transcription bias favouring English idiom cannot be entirely excluded, particularly for emotionally nuanced expressions, and the study relied entirely on adolescent self-report with no triangulation from caregivers or healthcare providers, whose perspectives might corroborate, complicate, or contradict adolescents’ accounts, particularly regarding adherence behaviour and family dynamics.

## Conclusion

This study provides rich insights into the lived psychological experiences of ALHIV in Ghana, highlighting both significant challenges and remarkable resilience. The findings underscore the need for comprehensive, integrated approaches to adolescent HIV care that address mental health, family systems, social support, and structural barriers. Critically, such approaches must be designed with, and remain sensitive to, the religious and cultural frameworks through which adolescents and their families interpret illness, stigma, and coping in this context, rather than applying standardised psychosocial models without cultural adaptation. By attending to these interconnected domains, healthcare systems can better support adolescents’ psychological well-being whilst improving engagement with life-sustaining treatment. Most importantly, the voices of these young people remind us that behind medical statistics are individuals navigating profound life challenges whilst demonstrating remarkable capacity for adaptation, meaning-making, and resilience.

## Data Availability

The original contributions presented in the study are included in the article/Supplementary material, further inquiries can be directed to the corresponding author.
