# Supplementary material for: Suicidal ideation, depressive experiences, and resilience among adolescents living with HIV in rural Ghana: a qualitative exploration of psychological experiences and coping
Source: Front Public Health. 2026 Oct 30;14:1972968. doi: 10.3389/fpubh.2026.1972968 (PMC13759360; doi:10.3389/fpubh.2026.1972968)
Supplement: Supplementary file 1 [file data_sheet_1.pdf]

Focus Group Discussion Guide

Study: Mental and Emotional Experiences of Adolescents Living with HIV/AIDS in Ghana

Opening Question

1. Please introduce yourself by your first name or a name you choose. Can you tell us a little about what your daily life looks like — school, home, and the things you enjoy doing?

[Purpose: Warm-up; establishes comfort and baseline normalcy before sensitive topics.

Main questions

2. Since you found out about your HIV status, how has it made you feel inside? Can you describe the emotions you experienced right after learning your status, and how those feelings have changed over time — if at all?

[Probes: Did you ever feel like giving up? Did you feel afraid about what might happen to you? Did you ever have thoughts of hurting yourself?]

3. Some young people living with HIV say they sometimes feel very sad, lose interest in things they used to enjoy, or find it hard to concentrate. Has anything like this happened to you? What do you think causes those feelings?

4. Have you ever been treated differently by people in your family, your community, your school, or your church or mosque because of your HIV status? Can you tell us about that experience and how it made you feel?

[Probes: Have family members ever blamed you or said hurtful things? Has a religious leader ever said anything about your condition?]

5. Have your peers or people in your community ever found out about your HIV status without you choosing to tell them? What happened, and how did that affect you?

6. How has living with HIV affected your relationships with friends, classmates, or people in your community? Are there places or situations you now avoid that you used to enjoy?

[Probes: Has your HIV status ever stopped you from going to school? Do you feel like you can move freely in your community?]

7. When you are feeling sad, worried, or overwhelmed about your health, is there someone you can talk to? Who gives you the most support, and what does that support look like?

[Probe: What happens on days when that person is not available?]

8. How do you feel about taking your medication every day? Are there times when you find it difficult to take your drugs — and if so, what makes it hard?

[Probes: Does getting to the hospital for your clinic days ever become a challenge? Do your thoughts about the future affect whether you take your medication?]

9. What has helped you keep going and stay strong despite all these challenges? What do you do — or who do you turn to when things feel very hard?

[Probes: Do you rely on prayer or your faith? Does your family play a role? Have you found any personal strategies that help you cope?]

Closing Question

10. If you could tell healthcare workers, teachers, or community leaders one thing about what young people living with HIV truly need — what would you want them to know or do differently?

[Purpose: Empowers participants; surfaces actionable insights for intervention design.]
